# Biopolymer-Based Films Reinforced with Fe_x_O_y_-Nanoparticles

**DOI:** 10.3390/polym14214487

**Published:** 2022-10-23

**Authors:** Johar Amin Ahmed Abdullah, Mercedes Jiménez-Rosado, José J. Benítez, Antonio Guerrero, Alberto Romero

**Affiliations:** 1 Departamento de Ingeniería Química, Escuela Politécnica Superior, Universidad de Sevilla, 41011 Sevilla, Spain; 2 Instituto de Ciencia de Materiales de Sevilla, Centro Mixto CSIC-Universidad de Sevilla, Calle Américo Vespucio 49, Isla de la Cartuja, 41092 Sevilla, Spain; 3 Departamento de Ingeniería Química, Facultad de Química, Universidad de Sevilla, 41012 Sevilla, Spain

**Keywords:** gelatin, cellulose acetate, chitosan, iron oxide nanoparticles, mechanical properties

## Abstract

Nowadays, natural polymer-based films are considered potentially environmentally friendly alternatives to conventional plastic films, due to many advantageous properties, including their easy processability, high flexibility, non-toxicity, low cost, high availability, and environmental safety. However, they are limited in their application by a number of shortcomings, including their high water solubility and vapor permeability as well as their poor opacity and low mechanical resistance. Thus, nanoparticles, such as green Fe_x_O_y_-NPs, can be used to overcome the drawbacks associated with these materials. Therefore, the aim of this study was to develop three different polymer-based films (gelatin-based, cellulose acetate-based and chitosan-based films) containing green synthesized Fe_x_O_y_-NPs (1.0% w/w of the initial polymer weight) as an additive to improve film properties. This was accomplished by preparing the different films using the casting method and examining their physicochemical, mechanical, microstructural, and functional characteristics. The results show that the incorporation of Fe_x_O_y_-NPs into the different films significantly enhanced their physicochemical, mechanical, and morphological properties as well as their antioxidant characteristics. Consequently, it was possible to produce suitable natural polymer-based films with potential applications across a wide range of industries, including functional packaging for food, antioxidants, and antimicrobial additives for pharmaceutical and biomedical materials as well as pesticides for agriculture.

## 1. Introduction

In the past few years, nanocomposites derived from biopolymer-based films have been widely investigated due to their potential as a technology for innovative design uses in eco-friendly food packaging [1]. Nevertheless, they have some disadvantages, such as their inadequate barrier and mechanical properties for resisting water vapor and oxygen. Therefore, nanoscale reinforcements are studied.

Films are thin (<1 mm), transparent, and stretchable plastics [2]. These materials provide excellent flexibility, are ideal for wrapping products of different sizes and shapes, and are typically produced from polyethylene (PE) or polypropylene (PP) [3]. These films possess several interesting properties, including their transparency (which allows visualizing the inside of the package), flexibility, adaptability, and impermeability (which provides a barrier that prevents air and moisture from flowing through, thereby protecting the content) [4]. All these properties are of interest for the food industry since films can prevent oxidation and reduction reactions and microbe interaction, thereby prolonging the shelf life of food products [5].

During the past few years, the demand for films has increased as a result of two critical factors: the importance of ensuring product safety during transport, thus increasing the requirement for packaging, and that the trend today is toward more attractive products, which increasingly utilize films for both hygienic and aesthetic purposes [6]. In this sense, food packaging is designed to protect it from several physical, chemical, and biological factors. Furthermore, it provides information to consumers regarding the ingredients, nutritional value, and safety of food products. Most polymeric materials are used in the food industry as packaging materials for direct contact with food [7]. However, these packaging materials are poorly biodegradable, creating an environmental problem. In this way, biopolymers, including polyesters, proteins, polysaccharides, and lipids, are utilized in order to satisfy industrial needs and consumer demands as well as to minimize their impact on the environment [8,9,10]. This has resulted in the development of a variety of natural biopolymer-based films, including those based on gelatin, cellulose, chitosan, or derivatives thereof, which have good biodegradability without toxic effects [11,12]. The raw materials such as gelatin, cellulose acetate, and chitosan possess properties which are potentially widely applicable in a variety of areas.

Gelatin is an edible biodegradable material derived from collagen, which is a protein-based natural biopolymer that is combined with functional ingredients (it is largely composed of amino acids such as glycine, proline, and hydroxyproline) to form efficient packaging materials [4]. It presents several advantages over other materials, including its ability to easily form effective films, excellent flexibility, appropriate gas barrier properties, low cost, and high reliability and availability [13,14]. On the other hand, it also exhibits high water solubility, high vapor permeability, and poor mechanical and thermal resistance [15,16]. Cellulose acetate is an ester of cellulose derived from the esterification of several cellulosic raw materials found in cotton, rice straw, sugarcane, wood, recycled paper, and bagasse [1,17]. It has several advantages, including an outstanding optical clarity, film formation at low temperatures, and chemical and thermal resistance. However, it has some weaknesses, such as a low dimension stability at high temperatures, stiffness, and the need for plasticizers for its industrial processing [1,17]. Likewise, chitosan is composed of poly (β-(1 → 4)-2-amino-2-deoxy-D-glucopyranose), which is a natural cationic polysaccharide derived from deacetylated chitin (*n*-acetylglucosamine and glucosamine units linked via β-(1 → 4)-glycosidic linkages). Chitin is very abundant, although less so than cellulose [18,19]. Nevertheless, it presents easy processability, non-toxicity, biocompatibility, biodegradability, genocompatibility, hemocompatibility, antibacterial activity, and environmental safety. In addition, it also has three functional groups (amine compounds (-NH_2_) and primary and secondary hydroxyl (OH) groups), which make it highly reactive. However, further investigation is needed to retain and/or improve such properties in chitosan-based materials [18].

These raw materials are widely used for a variety of industrial purposes; among them is food protection against light, drying, oxidation, water vapor, and chemical contamination through the incorporation of antioxidants, antimicrobials, nutrients, antifungals and flavors [20]. For cosmetic and hygienic applications, they are used as interfaces in shampoos, hair gels, and hair care products as well as in other cosmetic products [21,22,23,24]. Furthermore, they are of interest to the makers of biomedical and pharmaceutical applications, such as in anticancer, antidiabetic, antimicrobial, antioxidant and antihypertensive agents, wound care, tissue engineering and gene therapy [25,26]; they are also used as gelling agents for plasma expanders and as soft and hard polymer capsule fillers. Additionally, they are able to microencapsulate oils and pharmaceuticals and stabilize emulsions [27,28]. They also serve as protective coatings that increase a photograph’s shelf life and protective films to polarize LCD panels [24,29]. They are also used in other applications, such as glass frames, paints, and membranes for the treatment of water and fertilizers [22]. Regarding packaging functions, containment, protection, convenience, and communication are the most important factors. Thus, food packaging prolongs the shelf-life of packaged foods and maintains their safety. There are significant economic losses associated with lipid food deterioration during storage. Aside from microorganisms, the principal causes of spoilage are oxygen and chemical reactions. Food products deteriorate due to oxidation, which significantly limits their shelf life. Food oxidation may result in the loss of natural value (including proteins, soluble vitamins, and fatty acids), a reduction in energy content, the production of undesirable odors and flavors, and the degradation of pigment along with changes in color, all of which make the food less attractive to the consumers. Rancidity and the changes mentioned are the results of auto-oxidation, which involves a free radical chain mechanism. Due to this, food packaging manufacturers are continuously seeking effective methods of reducing the oxidation of lipids in food [7].

There are still numerous open investigations aimed at highlighting the properties of biobased films, including the incorporation of nano-scale materials as reinforcement fillers, which is one of the most significant methods [30]. In this way, several metal oxide nanoparticles, nanocelluloses, and nanoclays have been incorporated into biopolymer-based films [1,31,32,33,34,35,36,37]. In addition, the inclusion of certain nano-sized composites can replace select chemicals, thereby reducing the toxicity and cost of materials [38].

Regarding nanoparticles, magnetic iron oxide nanoparticles (Fe_x_O_y_-NPs) are extensively employed in biomedical applications owing to their magnetic properties, bioavailability, and biocompatibility [39]. Fe_x_O_y_-NPs inhibit the growth of several types of foodborne pathogens, including *Staphylococcus aureus* (S. au), *Escherichia coli* (E. col), and *Pseudomonas aeruginosa* (*p*. aeruginosa) [40]. Fe_x_O_y_-NPs produce reactive oxygen species (ROS) such as ^•^OH and ^•^O_2_^−^, causing damage to the DNA and proteins of bacteria and thus impairing mitochondrial function, without adversely affecting non-bacterial cells [2,18,20]. In addition, they are non-cytotoxic and nonhazardous at concentrations below 100 µg/mL [41] and can be used as an oral treatment for anaemia or iron deficiency [42]. In this context, this type of Fe_x_O_y_-NPs has been proposed as an appropriate additive to incorporate into films and enhance their antimicrobial properties. Nevertheless, Fe_x_O_y_-NPs can be synthetized by various methods, including traditional and green methods. Normally, the traditional method uses chemical reducing agents (i.e., NaOH) that can produce impurities and toxic residues in the final nanoparticles. The green method replaces this chemical reducing agent with a green one based on a polyphenol-rich extract. Thus, purer nanoparticles can be obtained without toxic residues. In addition, the election of the synthesis method can also condition the final properties of the obtained nanoparticles. In this way, the chemical production of Fe_x_O_y_-NPs leads to hazardous and agglomerated nanoparticles with lower stability. On the other hand, the green method achieves smaller sized, more stable, less agglomerated, purer, and less toxic iron oxide nanoparticles [43,44]. Nevertheless, several factors affect the green synthesis of nanoparticles, both in the pretreatment (such as the extract preparation, metallic salt, pH, and time of the reaction) and in the final treatment (such as the calcination temperature and time). In this way, the active polyphenols should not be degraded during the synthesis to maintain the maximum functionality of the final product. Therefore, it is novel to report an effective green method to synthesize iron oxide nanoparticles and subsequently incorporate them as natural additives into natural polymer-based films to overcome their deficiencies.

Thus, the main purpose of this study was to disperse these greenly synthetized nanoparticles at a concentration of 1.0% (*w*/*w* of the initial polymer weight) as an additive into three different films (gelatin-based, cellulose acetate-based, and chitosan-based films) to enhance their properties. To this end, films were processed by casting and were then characterized to compare their physicochemical, mechanical, microstructural, and functional properties.

## 2. Materials and Methods

### 2.1. Materials

Food gelatin (Ge, type B 200/220 g blooms, <10 ppm of sulfur dioxide) was provided by Manuel Riesgo, S.A. (Madrid, Spain), and cellulose acetate (CA, 39.8 wt% of acetyl content, Mn ca. = 30,000 g/mol, DS = 2.45) and chitosan (degree of deacetylation 98%, Mv = 1.61 × 105 g·mol^−1^) were supplied by Sigma Aldrich (Darmstadt, Germany). Acetone (CH_3_)_2_CO, Acetic acid CH_3_COOH 0.05 M, DPPH (2,2-diphenyl-1-picrylhydrazyl) and gallic acid (C_7_H_6_O_5_) were supplied by Sigma Aldrich (Darmstadt, Germany). The reagents used were all of analytical quality.

### 2.2. Nanoparticles Preparation

Green synthesized Fe_x_O_y_-NPs were obtained based on previous studies [45,46,47]. Briefly, they were synthesized by using colloidal precipitation method, mixing 20 mL of a polyphenol-rich solution (39 ± 2 mg GAE/g extract) extracted from *Phoenix dactylifera* L. with 20 mL of ferric trichloride hexahydrate FeCl_3_·6H_2_O (1M). In this case, NaOH 5 M was added dropwise to adjust the pH to 7.5, and the mixture was heated at 50 °C for 2 h under constant stirring (600 rpm). Thereafter, the precipitate was filtered, washed, and dried in an oven at 100 °C for 8 h. After that, it was subjected to a calcination treatment in a furnace at 200 °C for 2 h.

### 2.3. Film Processing Method

According to the method described in a previous study, biofilms were processed by casting [46]. Thus, 2% *w*/*v* of biopolymers (gelatin, cellulose acetate, and chitosan) were dissolved in distilled water, acetone, and 0.05 M acetic acid, respectively. Then, each solution was stirred at 60 °C for 2 h at 600 rpm. Subsequently, 1.0% *w*/*w* of Fe_x_O_y_-NPs were dispersed within the solutions using an ultrasound bath for 30 min (Ultrasounds, J.P Selecta, S.A., Barcelona, Spain) at 100 W sonication power and 50 Hz frequency. Finally, a constant volume of each solution (42.7 mL) was transferred into Teflon plates (7.6 cm in diameter), where they were dried at room conditions (22 °C and 35% relative humidity) for 72 h.

For the characterization, the films were peeled off carefully and stored in a desiccator. The reference films were processed without the incorporation of Fe_x_O_y_-NPs.

## 3. Characterization Technique

### 3.1. Nanoparticles Characterization

#### 3.1.1. X-ray Diffraction (XRD)

The green synthesized Fe_x_O_y_-NPs were conducted with an XRD pattern obtained using a Brand diffractometer (Bruker model D8 advance A25 diffractometer with Cu anode) to confirm their crystalline phase. The diffractograms were recorded at 2θ (°) = [15–70°]. According to previous studies, the Debye–Scherrer formula was used to calculate the size and crystallinity of the green synthesized Fe_x_O_y_-NPs [45,47].

#### 3.1.2. Transmission Electron Microscopy (TEM)

A TEM characterization was performed to evaluate the crystal systems as well as the sizes of the green synthesized Fe_x_O_y_-NPs. TEM images were observed at 200 kV using a Talos microscope (Talos S200 microscope, Thermo Fisher Scientific, Waltham, MA, USA). Then, the images of Fe_x_O_y_-NPs were labelled using Image-J 1.53 q free software to evaluate the average diameter size [45].

### 3.2. Physicochemical Properties of Films

#### 3.2.1. Water Solubility

The films were tested for water solubility according to previous studies with minor modifications [36,46,48,49]. Briefly, an initial weight (*W_i_*) of the samples (1 cm × 1 cm) was obtained after keeping them in a laboratory oven at 105 °C for 24 h. Afterwards, they were immersed in 25 mL of distilled water for 24 h, removed, and redried at 105 °C for 24 h in order to obtain the final dry weight (*W_f_*). Finally, the water solubility (*WS*%) was obtained using the following Equation (1):

(1) $$WS\left(\%\right)=\frac{w_{i}-w_{f}}{w_{i}}\cdot 100$$

#### 3.2.2. Contact Angle of Water (WCA) or Wettability of the Surface

The static water contact angle (WCA) was implemented to evaluate the hydrophobicity of the film surfaces using an optical tensiometer (Attension TL100, KSV, Helsinki, Finland) in the sessile drop mode. A horizontally levelled film (1 cm × 1 cm) was mounted on a movable stage and a ≈ 2 µL drop of DW (distilled water, Milli-Q grade) was placed onto the film surface using a µ-syringe. The drop contour was monitored for 20 s at 12 frames per second and the WCA was calculated on both sides. The frames that differed by more than 2° on the right and left sides were rejected. A minimum of five tests were conducted on each sample to ensure reproducibility.

#### 3.2.3. Water vapor permeability (WVP)

The measurement of the WVP of the different films was conducted based on the methodology defined in the ASTM E96 standard (ASTM, 2010) [50]. Thus, the films were preconditioned in a chamber at 25 °C and 50 ± 2% relative humidity (RH) for 48 h. Each film was placed by sealing an aluminium Payne-type test cup filled up to 2/3 of its internal volume with distilled water. Cups were then placed inside a dry chamber containing silica gel and purged with a gentle dry N_2_ stream that ensured virtually 0% RH. Dryness and temperature (22 ± 2 °C) in the chamber were continuously monitored with a combined sensor. The weight loss (0.1 mg precision) of the cups was monitored at regular periods until reaching a constant trend. The water vapor transmission rate (WVTR, g/h·m^2^·Pa) was determined as follows:

(2) $$WVTR=\frac{\alpha }{A}$$

where (α) is the linear regression slope (R^2^ > 0.998) of weight loss versus time and (A) is the permeation area of the cup.

(3) $$Permeance=\frac{WVTR}{\Delta p}=\frac{WVTR}{S\left(R_{1}-R_{2}\right)}$$

where (Δp) is the water vapor gradient between both sides of the film, which is calculated considering the water saturation vapor pressure (S) at the experiment temperature (2646 Pa) and the relative humidity at the water-exposed (*R*_1_) and chamber-exposed (*R*_2_) sides of the film, respectively, expressing it as fractions $\left(R_{1}-R_{2}=1-0\right)$.

(4) WVP=Permeance⋅film thikness

#### 3.2.4. Optical Properties (Light Transmission)

The transparency of each film was measured by UV-vis spectroscopy as described in a previous study [46]. Briefly, the transmittance of samples (1 × 2 cm^2^) was noted at 600 nm with a UV-vis spectrophotometer (Model 8451A, Hewlett Packard Co., Palo Alto, CA, USA) by transforming the absorbance values according to Lambert–Beer law. The results are expressed as transmittance percentage $T_{600}$%, and then the transparency (T) of the films was obtained according to the method described by Peighambardoust et al. [51]:

(5) $$T=\frac{-LogT_{600}}{t}$$

where $T_{600}$ is expressed as a fraction and refers to the amount of light the system can transmit and t represents the thickness of the film (mm). A higher transparency value indicates reduced light transmission across the film or a higher opacity.

### 3.3. Mechanical Properties

A static tensile test was conducted through a slight modification of the standard ISO 527–3:2019 [52] to assess the mechanical properties of the films. An axial force of increasing amplitude (at a rate of 10 mm/min) was applied to the samples until they broke, employing an MTS Insight 10 Universal Testing Machine (Darmstadt, Germany). In this test, the temperature and relative humidity (RH) were maintained at 22 °C and 35%, respectively. This test allows for the obtainment of the maximum stress (Ϭ_max_, MPa), strain at break (ε_max_, mm/mm), Young’s modulus or elasticity modulus (*Ε*, MPa), and toughness (kJ/m^3^).

### 3.4. Morphological Properties

The morphological properties and microstructures of neat and composite film surfaces were determined using scanning electron microscopy (SEM). Furthermore, the thicknesses of the samples were measured with ImageJ free software (1.53q; NIH, Bethesda, MD, USA). Before the observation, thin gold coatings were applied to the samples to improve their conductivity and, therefore, the image resolution. They were photographed in a Zeiss EVO microscope (Pleasanton, CA, USA) accelerated at 10 kV and magnified by 3000 X [53].

### 3.5. Functional Properties (Antioxidant Activity)

The efficacy of the films as antioxidants was evaluated according to Mehmood et al.’s protocol [36] but with a few modifications [46]. In brief, 1 mL of film-forming solution was admixed with 1 mL of methanolic solution of DPPH (40 ppm), followed by incubation for 0.5 h at room temperature. Each solution’s absorbance was measured at 517 nm using a spectrophotometer. For positive control, gallic acid was employed. The DPPH inhibition (*IP %*) could be calculated according to Equation (6).

(6) $$IP\;\left(\%\right)=\left(\frac{A-B}{A}\right)\times 100$$

where *A* is the absorbance of the uninhibited DPPH solution (without film solution as antioxidant agent) and *B* is the absorbance of the inhibited DPPH (with film-forming solution).

### 3.6. Statistical Analysis

All measurements in this study were conducted at least three times for each sample. A mean value and standard deviation are presented as a summary of the results, which were estimated using IBM SPSS software. Furthermore, an analysis of variance (one-way ANOVA) with 95% statistical confidence interval was used to estimate significant differences (*p* < 0.05).

## 4. Results

### 4.1. Nanoparticles Characterization

#### 4.1.1. XRD

**Figure 1:**
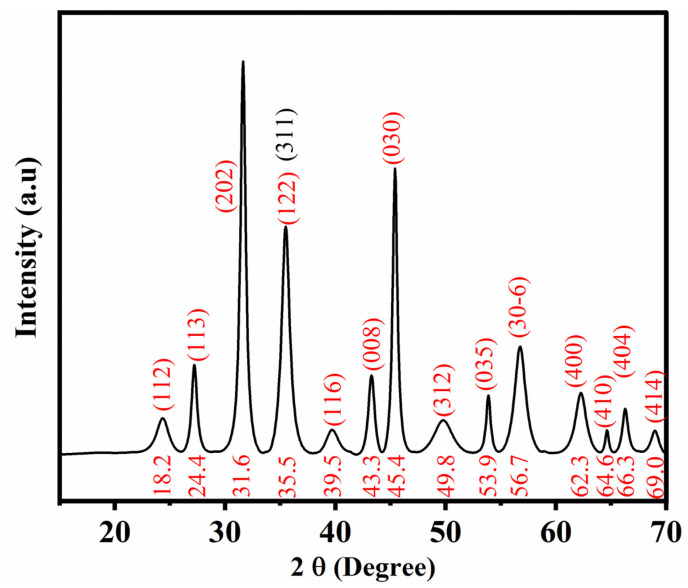
XRD spectra (JCPDS standard) of the green synthesized Fe_x_O_y_-NPs using aqueous extract of *Phoenix Dactylifera* L.

Figure 1 shows the X-ray diffractogram of the green synthesized Fe_x_O_y_-NPs (the magnetite phase is indicated by red 2θ ° and planes, whereas the hematite phase indicated by black 2θ ° and planes). The crystallite size, crystallinity, and phase composition were further investigated. The diffraction peaks of Fe_x_O_y_-NPs were attributed to ≈ 98.3% of magnetite in polycrystalline structures (29.3% cubic structures, 32.6% trigonal with a hexagonal axis, and 36.2% monoclinic) and cubic hematite (1.7%) (JCPDS n°. 00–210−1535, 00–152−8611, 00–153−2800, 00–900−7706, and 00–900−2673 standard iron oxide powder diffraction pattern) [54,55,56,57,58]. The mean size obtained through XRD was 10.2 ± 0.4 nm with 98.5% of crystallinity. These findings were further confirmed by TEM characterization. 

#### 4.1.2. TEM

**Figure 2:**
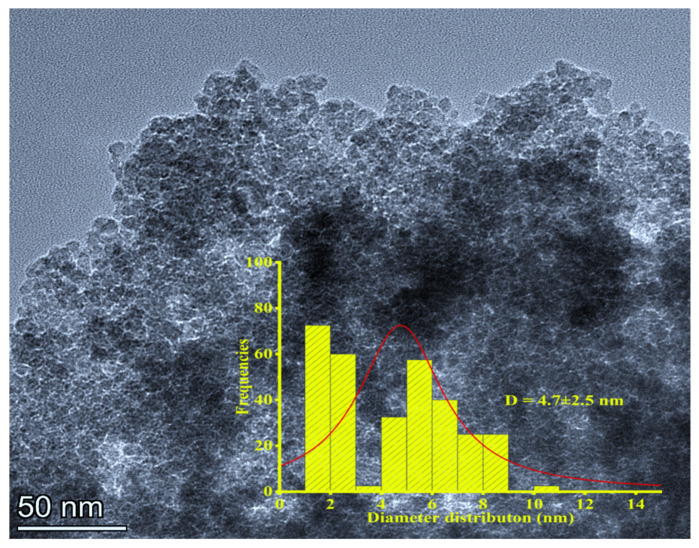
Transmission electron microscopy (TEM) of the green synthesized Fe_x_O_y_-NPs using aqueous extract of *Phoenix Dactylifera* L.

The morphology and the size distribution histogram (fitted by Lorentz curve) of FexOy-NPs are presented in Figure 2, with an average diameter (D = 4.7 ± 2.5 nm). As can be seen, the synthesized Fe_x_O_y_-NPs showed cubic and hexagonal structures, with well dispersed, extremely small nanoparticles. However, slight aggregation can be observed, which was probably caused by the competitive interactions between iron ions on the magnetite surface (Fe_3_O_4_-NPs) and the functional groups (phenolic compounds -OH^−^) of the extract [59,60]. The TEM results confirmed those obtained by XRD. 

### 4.2. Physicochemical Properties

#### 4.2.1. Water Solubility (WS)

A key food packaging parameter is water solubility (WS). Thus, insoluble films are required for better moisture resistance and product safety [36,49]. The WS% values of the neat and composite films are illustrated in Table 1. Thus, the neat gelatin-based film was the most water-soluble (WS = 87.9%), which is probably due to gelatin hydrophilicity [61]. Nevertheless, the incorporation of Fe_x_O_y_-NPs significantly enhanced the water resistance of the films, causing a WS reduction ranging from 15% for the gelatin composite to 38% for the cellulose acetate composite. These results are consistent with that of Hosseini et al. (2015), who concluded that the incorporation of chitosan nanoparticles (6% *w*/*w*) into the gelatin matrix reduced the solubility from 71.8 to 62.6% [49]. However, the literature contains some contradictory results; for example, the incorporation of capsaicin- ${\mathrm{Fe}}^{+3}$ doped hollow metal-organic frameworks (cap-${\mathrm{Fe}}^{+3}$-HMOF-5) into gelatin/chitosan films increased the water solubility from 45.7 to 58.4%, according to Xiaojun et al. (2020) [62]. Neat acetate cellulose-based films exhibited much lower solubility (WS = 8.5%) due to their insolubility in water [63]. Furthermore, the incorporation of Fe_x_O_y_-NPs enhanced this insolubility (lowest WS = 5.3%, Table 1). A similar decrease in water solubility was also reported when cellulose nanofibers (C-NFs) were incorporated into cellulose acetate-based films [17]. Finally, the water solubility of the chitosan-based film was reduced from 18.1 ± 1.5 to 15.4 ± 0.8% when the Fe_x_O_y_-NPs were incorporated. A similar decrease in solubility was reported with regard to the incorporation of SiO_2_-NPs-GA into chitosan films [64]. This could be due to the hydrophobic nature of chitosan. Generally, the water absorption reduction that was observed in all the films could be attributed to the formation of hydrogen bonds between the nanoparticles and polymer chains [61]. Thus, the incorporation of hydrophobic materials into polymer results in an increase in hydrophobic compounds, thereby reducing their solubility [65]. In addition, the high crystallinity of nanoparticles/nanofillers (in this work, 98.5%) was also reported as a contributing factor in improving water resistance [17]. Nevertheless, the water solubility of the films characterized in this work is higher than those obtained by a commercial material such as polyethylene. Regarding the different neat films, it is obvious that their water solubility was affected by their hydrophilicity/hydrophobicity characteristics, which was further confirmed by water contact angle measurements.

#### 4.2.2. Water Contact Angle (WCA) 

**Figure 3:**
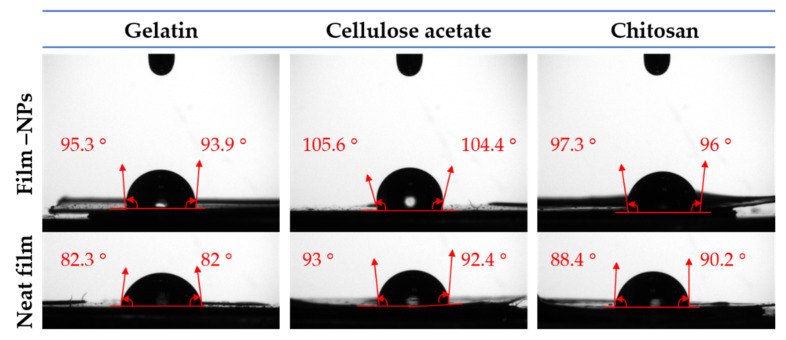
The water contact angles photographs of the different films (gelatin-based, cellulose acetate-based, and chitosan-based composite films) with Fe_x_O_y_ (1.0% *w*/*w*). Neat films (gelatin-based, cellulose acetate-based, and chitosan-based films) without Fe_x_O_y_ nanoparticles incorporated were included as references.

In the design of food packaging films, the WCA plays an important role, indicating the hydrophilic/hydrophobic character or the surface’s wettability [51]. Hydrophilicity and hydrophobicity are related to WCA values, thus the smaller the angle (acute angle inside the drop), the greater the hydrophilicity, whereas the greater the angle (obtuse angles or over 90 °), the greater the hydrophobicity [51,66]. The water contact angles of neat films and composite films are illustrated in Figure 3, and their corresponding values are shown in Table 1. The WCA values varied with the polymer’s nature and were also affected by nanoparticle incorporation. For gelatin, a mild hydrophilic behavior was observed (WCA = 82.2 °), despite the presence of hydrophilic chains in the polymer framework [67]. This behavior can be attributed to the orientation of the hydrophobic groups at the interface between gelatin and air during gelation or solvent evaporation. Therefore, hydrophilic groups (carboxyl and amino) tend to form internal hydrogen bonds, while hydrophobic groups (aryl and aliphatic) tend to form external hydrogen bonds. This indicates a relatively non-wettable surface of neat gelatin caused by the presence of nonpolar protein segments that are exposed at the exterior surface of the film upon the removal of the solvent [51]. The incorporation of Fe_x_O_y_-NPs increased the WCA to 94.6°, which may indicate the higher hydrophobicity of iron nanoparticles, this value is close to commercial material value. Iron ions form a crosslink complex that bonds to macromolecules through multiple means, such as hydrophobic interactions and hydrogen and ionic bonding [68]. These findings are consistent with those of previous studies [67,69]. Neat cellulose acetate-based films exhibited a moderate water contact angle value (WCA = 92.7°) due to the hydrophobicity of cellulose acetate [70,71]. The incorporation of hydrophobic Fe_x_O_y_-NPs enhanced the hydrophobicity of the cellulose acetate-based nanocomposite films by increasing the WCA to 105 °. This value is significantly higher than that of commercial material. It has been reported that hydrophobic nanoparticles increase water contact angles when exposed to membrane surfaces [66]. Similar findings were reported for the incorporation of Cu-NPs into cellulose acetate films [72] and silica particles into cellulose acetate/chitosan films (CA/CS-Si) [73]. Likewise, neat chitosan appeared to be relatively hydrophobic, with a WCA = 89.4°, most likely due to chitosan’s hydrophobic backbone [74]. The Fe_x_O_y_-NPs incorporated into the chitosan-based films increased the hydrophobicity of the film surface to 96.7°. Similarly, other nanoparticles were incorporated into chitosan-based films with similar results [75]. Moreover, the chemical effect of Fe_x_O_y_-NPs on the hydrophobicity of the composites can be complemented with the increase in surface roughness caused by nanoparticles (see Section 4.4.), and as previously reported for other blends [76].

#### 4.2.3. Water Vapor Permeability (WVP)

Water vapor permeability in packaging materials is causing concern among several food industries. An evaluation of the effect of nanoparticles on composite films was conducted through the determination of the water vapor transmission rate (WVTR). Results for the WVTR, permeance, and WVP are shown in Table 1. In all cases, the incorporation of Fe_x_O_y_-NPs resulted in a significant reduction in water vapor transmission rate values and, therefore, in the water vapor permeability of the films. The mean WVP of the gelatin-based nanocomposite film was reduced by approximately 8% with the mean WVP of the neat gelatin being reduced from 2.7 ± 0.03 to 2.5 ± 0.08 (g·m/h·m^2^·Pa) ×$10^{-6}$. This may be due to the creation of well-interconnected three-dimensional networks [77]. The dispersion of nanoparticles (NPs) in the polymer matrix may create twisted pathways, which obstruct and delay the passage of water molecules across the film matrix [49], together with the restriction of the movement of protein chains in the gelatin framework, hindering the travelling of water molecules [75,78]. Other studies have indicated that the hydrophobic/hydrophilic balance of the filler might be marginally affected by switching the cation. Due to strong interactions between the nanoparticles and polymer chains, a number of hydrophilic groups may be consumed, resulting in a reduction in water transmission [79]. On the other hand, the WVP of the cellulose acetate-based film enforced with Fe_x_O_y_-NPs was reduced by about 13% with respect to the WVP of the neat cellulose film, which was 6.7 ± 0.10 (g·m /h·m^2^·Pa) ×$10^{-6}$. Likewise, the WVP of chitosan embedded with Fe_x_O_y_-NPs decreased by 29% when compared with the neat chitosan film (4.3 ± 0.04 g·m/h·m^2^·Pa ×$\;10^{-6}$, Table 1), which may be due to an enhancement in polymer matrix hydrophobicity [80]. Nevertheless, the water vapor permeability of composite films is affected by multiple factors, including hydrophobicity/hydrophilicity, thickness, roughness, compaction, particle size, crystallinity, distribution, and orientation [9,67,81]. In general, nanoparticles contribute to a reduction in the water vapor permeability of a film by reducing the number of free hydroxy groups or by enhancing the hydrophobicity and crystallinity and, therefore, improving the moisture resistance of the film matrix [12,82]. Nevertheless, the resulting permeability is superior to that of the commercial material.

#### 4.2.4. Optical Properties

**Figure 4:**
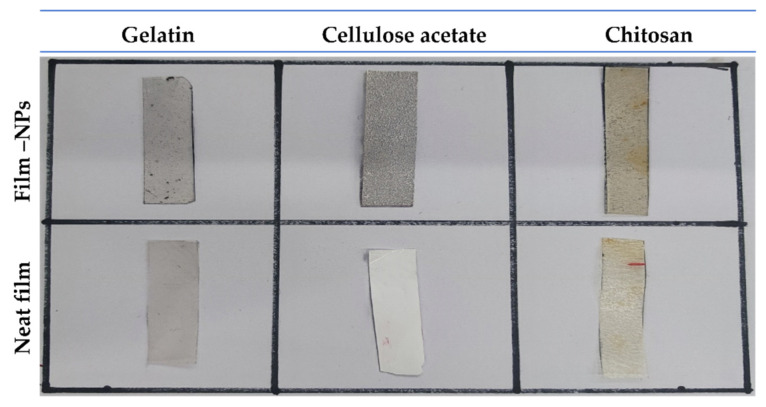
Photographs of the different films (gelatin-based, cellulose acetate-based, and chitosan-based composite films) with Fe_x_O_y_ (1.0% *w*/*w*). Neat films (gelatin-based, cellulose acetate-based, and chitosan-based films) without Fe_x_O_y_ nanoparticles incorporated were included as references.

Photographs of the different films are provided in Figure 4. The transmittance ($T_{600}$%) and transparency values (T) of neat films and composite films are presented in Table 1. The incorporation of Fe_x_O_y_-NPs into the films significantly decreased the transmittance values and increased the transparency index. The transmittance of neat gelatin decreased from 61% to 41% when Fe_x_O_y_-NPs (1%) were incorporated, where the transparency was increased from 2.41 to 3.65. This could be attributed to the increase in the solid composition (nanoparticles) in the polymer chains, thereby restricting their mobility. In this way, the dispersion of nanofillers into the polymer chains may fill up vacant spaces and block light from passing through the film. Several studies reported similar results when metallic oxide nanoparticles were added to gelatin-based films [31,36,51,83,84]. Regarding transparency, cellulose acetate-based films were determined to be the least transparent of the series. Thus, cellulose acetate without nanoparticles displayed a transmittance of 14.89 ± 1.3%, and with the further addition of Fe_x_O_y_-NPs, the transmittance was reduced to 10.35 ± 0.8%. Regarding the thickness of the film, the transparency value of the neat cellulose acetate-based film increased from 5.55 to 6.12 (a significant increase with *p* < 0.05). This may be the result of the increase in solid material within the polymer chains. Likewise, neat chitosan also demonstrated an intermediate transmittance, which was reduced by filling up the free space formed during film formation. As a result, its transparency value (opacity) increased from 4.76 to 5.79 (Table 1, *p* < 0.05).

### 4.3. Mechanical Properties

**Figure 5:**
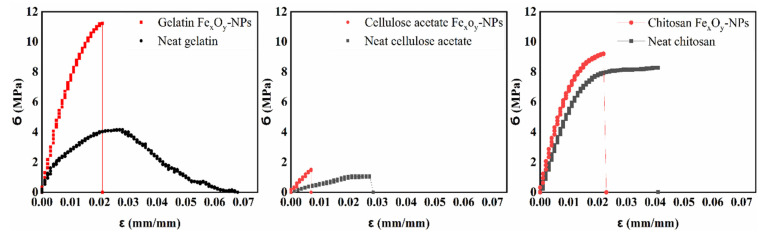
Tensile test profile of the different films (gelatin-based, cellulose acetate-based, and chitosan-based composite films) with Fe_x_O_y_ (1.0% *w*/*w*). Neat films (gelatin-based, cellulose acetate-based, and chitosan-based films) without NPs incorporated were included as the reference system.

The tensile profile of the different neat and composite films is shown in Figure 5. The mechanical parameters of the different films are also represented in order to facilitate the comparison of the systems (Table 2).

As shown in Figure 5, all neat film systems were characterized by a short elastic zone and then a longer plastic region. This plastic zone was drastically reduced when the Fe_x_O_y_-NPs were incorporated into the film. The composites became more brittle by increasing the maximum stress (Ϭ_max_, MPa) and Young’s modulus (*Ε*, MPa) and by decreasing the ε_max_. The Ϭ_max_ and *Ε* of neat gelatin increased from 4.2 ± 0.1 to 11.1 ± 0.2 MPa and from 71.7 ± 17.8 to 555.6 ± 17.8 MPa, respectively, due to the incorporation of Fe_x_O_y_-NPs. Similarly, these parameters were found for neat cellulose acetate and neat chitosan (Table 2).

On the other hand, the (Ϭ_max_, MPa) and (*Ε*, MPa) of the neat cellulose acetate-based film were the lowest (Table 2), which may be due to the fact that cellulose acetate requires a plasticizer [1,17]. Similar findings were reported by Yadav (2018), who studied the incorporation of magnetite (Fe_3_O_4_-NPs) into cellulose-based films [85]. The addition of immiscible particles may lead to non-homogeneous networks in the films, thereby limiting their extensibility [86]. Through NP-protein hydrogen bonding, nanoparticles may enhance the mechanical resistance of polymer films [36].

The strain at break (ε_max_) was reduced when Fe_x_O_y_-NPs were incorporated into the systems. This is because nanoparticle incorporation stiffens films due to the increase in solid material [36]. The incorporation of Fe_x_O_y_-NPs into polymer chains also reduces the cohesion forces between the chains, thereby reducing the strain at break [87]. Moreover, these results may also be attributed to the type and size of nanoparticles, as reported in a previous study [46]. Consequently, smaller nanoparticles could result in greater maximum stress and Young’s modulus, since better interconnections in the structure and a stronger network between nanoparticles and polymer chains were generated [88]. Furthermore, the presence of immiscible particles leads to less homogeneous structures, which in return reduces the connectivity of the polymer networks [89,90]. In addition, the calculated toughness was reduced when Fe_x_O_y_-NPs were incorporated into the cellulose and chitosan systems due to the lower deformation, but there was no significant difference observed with regard to the gelatin system (Table 2).

### 4.4. Morphological Properties

#### Scanning Electron Microscopy (SEM)

**Figure 6:**
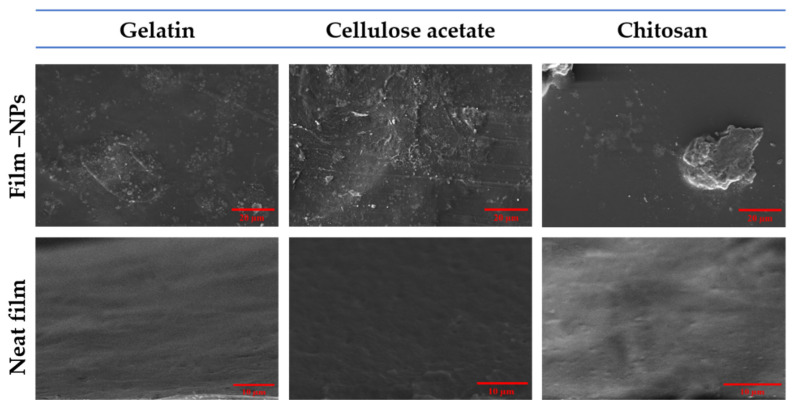
Scanning electron microscopy (SEM) images for the upper surfaces of the different films (gelatin-based, cellulose acetate-based, and chitosan-based composite films) with Fe_x_O_y_ (1.0% *w*/*w*). Neat films (gelatin-based, cellulose acetate-based, and chitosan-based films) without NPs incorporated were included as the reference system.

The surface morphologies of the different neat and composite films are shown in Figure 6. The neat film surface textures were smooth and homogeneous. In contrast, the incorporation of Fe_x_O_y_-NPs produced irregularities and an increment in terms of plasticizer. This could be attributed to the different characteristics of the nanoparticles, such as granulation, size, dispersion, and aggregation on the surface during the process of solvent evaporation [51,67]. The nanoparticles were uniformly distributed, with slight aggregation in the gelatin and cellulose systems, whereas agglomeration was observed in the chitosan system. The aggregation of nanoparticles occurs as a result of charge interactions between the functional groups (phenolic compounds) on the surface of the Fe_x_O_y_-NPs and the biopolymer chains in the films, altering their structure [91,92]. The aggregation of nanoparticles may also be affected by the solvent’s nature; thus, the rate of exchange between solvents and non-solvents can be delayed or accelerated [93]. Furthermore, the aggregation of metal oxide nanoparticles became greater as the content of metal oxide nanoparticles increased [94]. Therefore, enhancing the surface roughness of polymer surfaces is of continued interest since it can either favor or inhibit macromolecule adsorption or biofilm formation [95].

### 4.5. Functional Properties (Antioxidant Activity)

The antioxidant activity of neat and composite films was evaluated against DPPH radicals. Table 2 presents the DPPH inhibition percentage *(IP%)*. Accordingly, an increase in antioxidant activity was recorded in the presence of Fe_x_O_y_-NPs, which may be due to the fact that these nanoparticles grant antioxidant properties to the systems in which they are present [36,96]. Regarding the different neat films, gelatin exhibited the lowest inhibition of DPPH (24.9 ± 1.5%, Table 2). However, this result is about four times higher than that reported by Zaffar et al. (2020) for gelatin-based films (5% *w*/*v*; 5% of glycerol *w*/*w*) under similar conditions. They found that the gelatin without nanoparticles exhibited roughly 6.32% of DPPH inhibition, and when they incorporated iron oxide nanoparticles (6.5 ± 3.0 nm) in different concentrations (5, 10, 15, and 20% *w*/*w*) into gelatin films, the corresponding DPPH inhibitions were roughly 20.2, 30,9, 42.2, and 55.1%, respectively [36]. In this study, the DPPH inhibition by the gelatin embedded with Fe_x_O_y_-NPs (1% *w*/*w*) was much higher (roughly 78.1%). Meanwhile, the inhibition of DPPH by neat cellulose acetate increased to 64.7 ± 0.9% with the incorporation of Fe_x_O_y_-NPs, versus the 37.8 ± 2.5% inhibition for neat cellulose acetate. Nevertheless, some studies have reported negligible antioxidant activity for cellulose derivatives. For example, Swarup et al. (2020) reported a DPPH inhibition of 1.9% for neat carboxymethyl cellulose-based films. In contrast, when curcumin (1 wt%) and zinc oxide (1 wt%) were incorporated, DPPH inhibition increased to 3.7 and 40.2%, respectively [97]. Likewise, the neat chitosan showed the highest antioxidant activity among all the neat films, with a DPPH inhibition of 42.8 ± 1.7%, whereas the incorporation of Fe_x_O_y_-NPs increased this percentage to 88.6 ± 1.4%. The higher level of DPPH inhibition by neat chitosan is attributed to the presence of free amino functional groups, which interact with free radicals to produce macromolecular free radicals and highly stable ammonium groups [64]. Some results in the literature found that neat chitosan-based film (2% *w*/*v* in 1% of acetic acid) inhibited DPPH free radicals by 18%, whereas DPPH inhibition increased to 92% in the presence of green SiO_2_-NPs (8 mg/mL) [64]. These results could indicate the effect of the nature and concentration of the solvent used to prepare the films as well as the nanofiller concentrations [64,93]. The increase in DPPH inhibition in the presence of Fe_x_O_y_-NPs is believed to be due to the presence of higher concentrations of antioxidants in these particles, derived from polyphenols during their synthesis, as reported in previous work [46]. Thus, the FexOy-NPs were green synthesized and showed advantages in both the synthesis process and the generation of nanoparticles, which have greater utility in future applications and reduce toxic waste and costs. In addition, gallic acid as positive control exhibited *IP %* = 95%. The antioxidant properties of the green synthesized Fe_x_O_y_-NPs benefit the antimicrobial properties already demonstrated in a previous study [46].

## 5. Conclusions

The green synthesis of iron oxide nanoparticles using *Phoenix dactylifera* L. demonstrated high efficiency in obtaining well-dispersed nanostructures with higher crystallinity and effective magnetite (>98%) to be used in a wide range of applications. Food packaging is an ideal application for iron oxide nanoparticles due to their many features. Currently, food packaging materials suffer from a variety of deficiencies, including poor physicochemical, mechanical, and functional properties.

The Fe_x_O_y_ nanoparticles obtained in this study demonstrated great potential to improve the properties of natural polymer-based films of gelatin, cellulose, and chitosan. Thus, the Fe_x_O_y_ nanoparticles used in this study enhanced the hydrophobicity of the films, thereby reducing their solubility in water and their water vapor permeability, in addition to increasing the water contact angle. Moreover, the incorporation of Fe_x_O_y_-NPs increased film thickness and solid material content, which hinders the passage of light, thus increasing the opacity of the films. In addition, the stiffness of the samples was increased by the incorporation of these nanoparticles. The use of green synthesized iron oxide nanoparticles improved the antioxidant properties of the fabricated films. Thus, with 1% of Fe_x_O_y_-NPs (*w*/*w*), the inhibition of DPPH free radicals ranged from 65–89%. 

Considering all these attributes, natural polymer-based films with incorporated Fe_x_O_y_-NPs have potential for use as functional packaging materials with antioxidant and antimicrobial capabilities. Nevertheless, further research is needed to assess the potential migration of nanoparticles that could be an issue in healthcare and food applications.

## Figures and Tables

**Table 1:** Physicochemical and optical values of the different films embedded with Fe_x_O_y_ nanoparticles (1.0 %). Neat films (gelatin-based, cellulose acetate-based, and chitosan-based films) without Fe_x_O_y_ nanoparticles incorporated were used as references. The parameters of a commercial material based on polyethylene were included as reference. Different superscript letters (a–f) for each column indicate heterogeneity of variances (*p* < 0.05).

Sample	WS (%)	WCA (°)	WVTR (g/h·m^2^)	Permeance (g/h·m^2^·Pa) ×10^−2^	WVP (g·m/h·m^2^·Pa) ×10^−6^	T_600_ (%)	T
Commercial reference	<1%	96	4–23	1	0.1 ^8^	-	-
Gelatin Fe_x_O_y_-NPs	66.7 ± 1.3 ^b^	94.6 ± 1 ^c^	62.2 ± 2.3 ^e^	2.4 ± 0.07 ^e^	2.5 ± 0.08 ^f^	41.2 ± 1.3 ^b^	3.65 ± 0.01 ^e^
Neat gelatin	87.9 ± 2.2 ^a^	82.2 ± 0.2 ^f^	80.3 ± 1.3 ^d^	3.0 ± 0.04 ^d^	2.7 ± 0.03 ^e^	61.0 ± 1.1 ^a^	2.41 ± 0.01 ^f^
Cellulose acetate Fe_x_O_y_-NPs	5.3 ± 0.9 ^f^	105 ± 0.8 ^a^	121.8 ± 1.0 ^b^	4.6 ± 0.07 ^b^	6.7 ± 0.10 ^b^	10.35 ± 0.8 ^f^	6.12 ± 0.04 ^a^
Neat cellulose acetate	8.5 ± 1.2 ^e^	92.7 ± 0.4 ^d^	129.5 ± 2.3 ^a^	4.9 ± 0.03 ^a^	7.9 ± 0.06 ^a^	14.89 ± 1.3 ^e^	5.55 ± 0.01 ^c^
Chitosan Fe_x_O_y_-NPs	15.4 ± 0.8 ^d^	96.7 ± 1.0 ^b^	88.1 ± 1.3 ^c^	3.3 ± 0.05 ^c^	3.0 ± 0.04 ^d^	29.56 ± 1.7 ^d^	5.79 ± 0.01 ^b^
Neat chitosan	18.1 ± 1.5 ^c^	89.3 ± 1.8 ^e^	116.6 ± 1.6 ^a^	4.1 ± 0.04 ^a^	4.3 ± 0.04 ^c^	34.4 ± 1.2 ^c^	4.76 ± 0.01 ^d^

**Table 2:** Results for thickness, mechanical parameters (Ϭ_max_, ε_max_, *Ε*, and toughness), and antioxidant activity values (DPPH *IP (%)*) of the different films embedded with Fe_x_O_y_ nanoparticles (1.0%). Neat films (gelatin-based, cellulose acetate-based, and chitosan-based films) without Fe_x_O_y_-NPs blended were used as references. Different superscript letters (a–f) for each column ascertain heterogeneity of variances (*p* < 0.05).

Sample	Thickness (µm)	Ϭ_max_ (MPa)	ε_max_ (mm/mm)	*Ε* (MPa)	Toughness (kJ/m^3^)	DPPH *IP (%)*
Gelatin Fe_x_O_y_-NPs	105.6 ± 1.7 ^c^	11.1 ± 0.2 ^a^	0.02 ± 0.001 ^d^	555.6 ± 17.8 ^a^	150 ^b^	78.1 ± 1.1 ^b^
Neat gelatin	89.2 ± 0.8 f	4.2 ± 0.1 ^d^	0.06 ± 0.012 ^a^	71.7 ± 12.9 ^e^	150 ^b^	24.9 ± 1. 5 ^f^
Cellulose acetate Fe_x_O_y_-NPs	161.1 ± 3.4 ^a^	1.5 ± 0.1 ^e^	0.01 ± 0.001 ^e^	150.3 ± 5.1 ^d^	10 ^e^	64.7 ± 0.9 ^c^
Neat cellulose acetate	148.9 ± 2.3 ^b^	1.0 ± 0.1 ^f^	0.03 ± 0.001 ^c^	33.3 ± 2.2 ^f^	20 ^d^	37.8 ± 2.5 ^e^
Chitosan Fe_x_O_y_-NPs	97.3 ± 1.5 ^d^	9.2 ± 0.2 ^b^	0.02 ± 0.001 ^d^	460.4 ± 13.0 ^b^	140 ^c^	88.6 ± 1.4 ^a^
Neat chitosan	91.4 ± 0.6 ^e^	8.2 ± 0.1 ^c^	0.04 ± 0.001 ^b^	205.0 ± 2.6 ^c^	270 ^a^	42.8 ± 1.7 ^d^

## Data Availability

The data presented in this study are available on request from the corresponding author.
